# Supplementary material for: Endosomal chemokine receptor signalosomes regulate central mechanisms underlying cell migration
Source: eLife. 2025 Feb 24;13:RP99373. doi: 10.7554/eLife.99373 (PMC11850004; doi:10.7554/eLife.99373)
Supplement: Figure 4—source data 1. [file elife-99373-fig4-data1.pdf]

HEK293-CCR7-APEX2

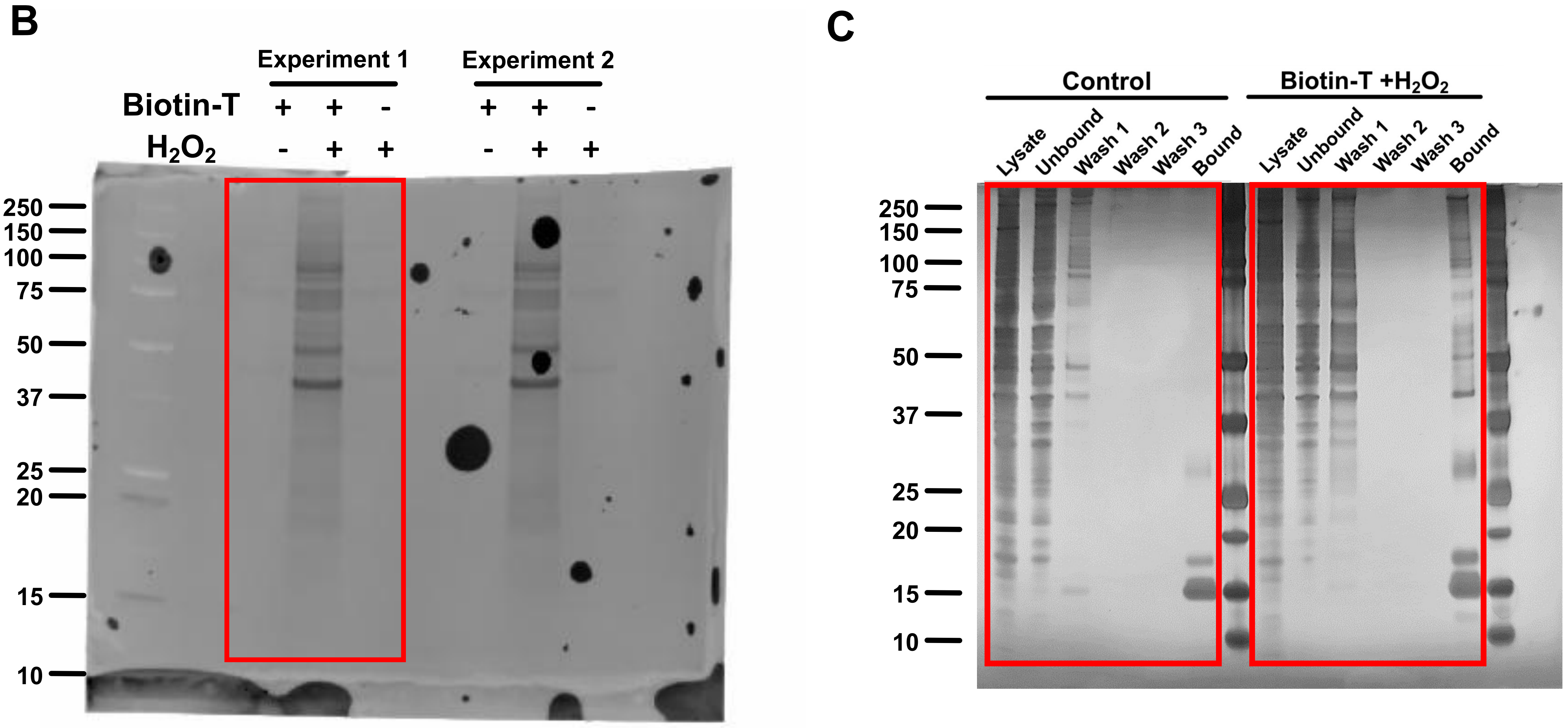

WB: Streptavidin-Alexa488
Marker: Precision Plus Protein Dual Color Standards (Bio-Rad)

Silver stain
Marker: Precision Plus Protein Dual Color Standards (Bio-Rad)
