# Supplementary figures and images for: Endosomal chemokine receptor signalosomes regulate central mechanisms underlying cell migration
Source: eLife. 2025 Feb 24;13:RP99373. doi: 10.7554/eLife.99373 (PMC11850004; doi:10.7554/eLife.99373)

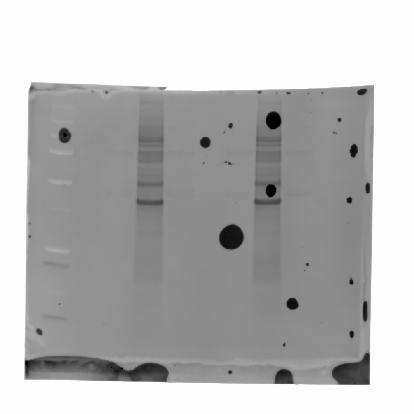

Supplement: Figure 4—source data 2. [file elife-99373-fig4-data2.zip › Streptavidin-Alexa488 blot.tiff]

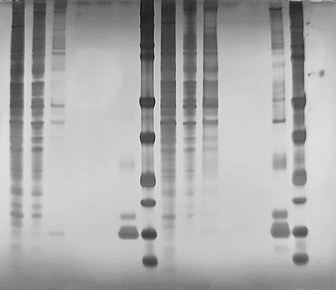

Supplement: Figure 4—source data 2. [file elife-99373-fig4-data2.zip › Silver stain.tiff]

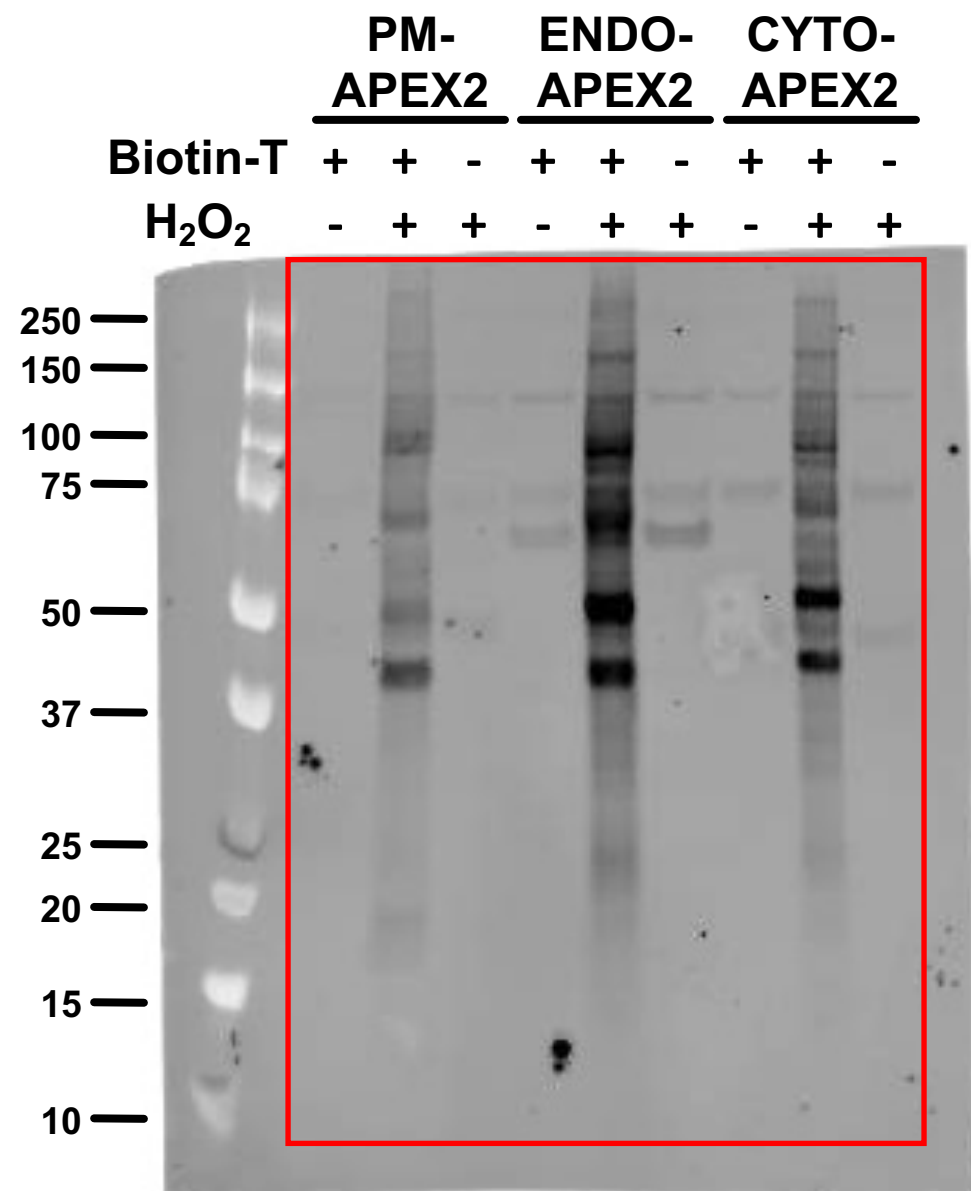

WB: Streptavidin-Alexa488

Marker: Precision Plus Protein Dual Color Standards (Bio-Rad)

Supplement: Figure 5—figure supplement 2—source data 1. [file elife-99373-fig5-figsupp2-data1.pdf]

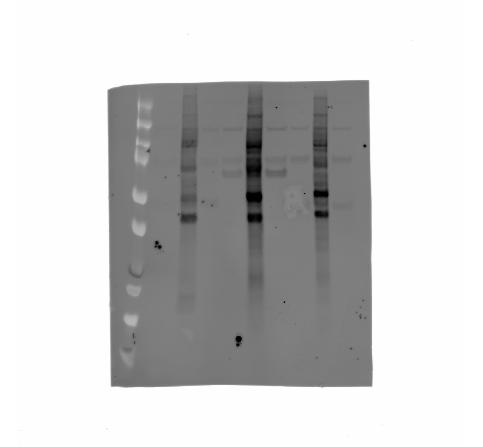

Supplement: Figure 5—figure supplement 2—source data 2. [file elife-99373-fig5-figsupp2-data2.zip › Streptavidin-Alexa488 blot.tiff]

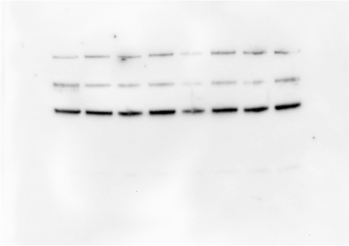

Supplement: Figure 6—figure supplement 1—source data 2. [file elife-99373-fig6-figsupp1-data2.zip › Beta-tubulin blot.tiff]

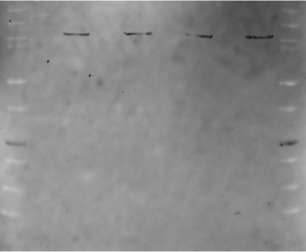

Supplement: Figure 6—figure supplement 1—source data 2. [file elife-99373-fig6-figsupp1-data2.zip › HA blot.tiff]
