# Supplementary material for: Endosomal chemokine receptor signalosomes regulate central mechanisms underlying cell migration
Source: eLife. 2025 Feb 24;13:RP99373. doi: 10.7554/eLife.99373 (PMC11850004; doi:10.7554/eLife.99373)
Supplement: Figure 6—figure supplement 1—source data 1. [file elife-99373-fig6-figsupp1-data1.pdf]

HEK293-CCR7 cells transfected with either pcDNA3.1 (–) or HA-Dynamin1-K44A (K44A)

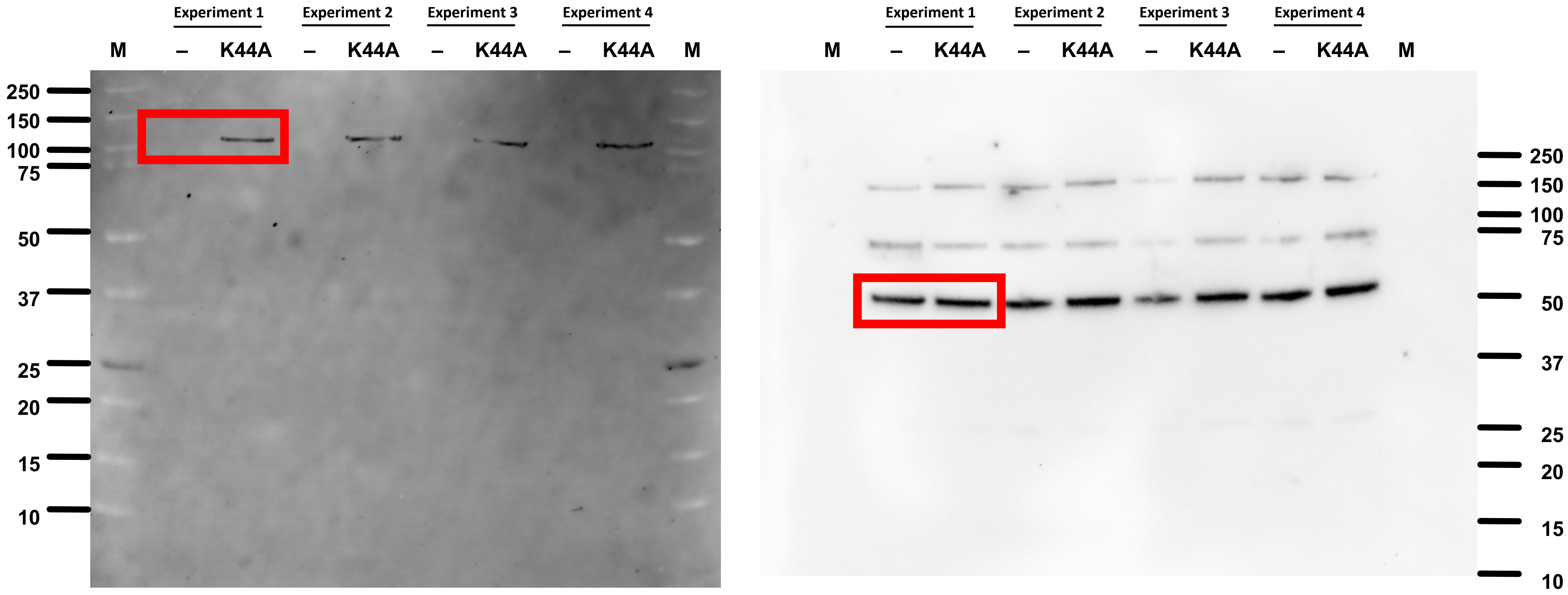

WB: Anti-HA (left) and anti-β-tubulin (right)
Marker: Precision Plus Protein Dual Color Standards (Bio-Rad)
